# Human capital-based four-factor asset pricing model: An empirical study from Pakistan

**DOI:** 10.1016/j.heliyon.2023.e16328

**Published:** 2023-05-16

**Authors:** Naveed Khan, Hassan Zada, Shakeel Ahmed, Fayaz Ali Shah, Shahid Jan

**Affiliations:** a Faculty of Management Sciences, HITEC University, Taxila 47080, Pakistan; b Department of Management Sciences, Shaheed Zulfikar Ali Bhutto Institute of Science and Technology (SZABIST), Islamabad 44000, Pakistan; c Faculty of Management Sciences, Islamia College University, Peshawar 25120, Pakistan

**Keywords:** Asset pricing, Human capital, Four-factor model, Labor income, Pakistan stock Exchange

## Abstract

This study aims to extend the Fama-French three-factor model by including human capital as a fourth factor. For this purpose, we have collected data from 164 non-financial firms from July 2010 to June 2020. To evaluate the validity and applicability of our augmented human capital-based four-factor model, we apply the two-pass time series regression proposed by Fama-Macbeth (1973). We find that small firms outperform big firms, value stocks firms outperform growth stocks firms, and low-labor-income firms outperform high-labor-income firms. The augmented human capital-based four-factor model is valid and applicable in the context of the Pakistan equity market. The empirical results motivate academia and all investors to consider human capital in investment decisions.

## 1 Introduction

The rapid advancements in emerging markets has gained the insight of potential investors and academicians to access the risk inherent in these markets. Such rapid growth and development of emerging market questions mark the unknown factor that is different from developed capital markets. In the recent past, market anomalies in the assets pricing framework have been challenged [1]. One of the most contentious areas of finance is asset pricing. This is due to investors' desire to determine the intrinsic value of financial assets for making investment decisions [2]. Similarly [3], documents that there are two steps involved in selecting financial securities. The first step is observation, which relies on the available information and opinions about the performance of future stocks. While the second step experiences when the investors presume to create a portfolio to maximize the expected stock returns with undiversified risk.

In financial economics, there is a question about estimating asset returns. For this purpose, there are two alternatives available, the first being the single-factor model or CAPM, which is a capital asset pricing model proposed by Ref. [4], later on by Ref. [5], and after it proposed by Ref. [6], independently of one another. The second is the multifactor models of [7], who proposed the APT (Theory of Arbitrage Pricing)- a multifactor model for evaluating stock returns. This theory does not mention the countable factors nor addresses portfolio efficiency. According to Ref. [8], firms with higher price-to-earnings ratios (P/E ratios) generate noticeably higher returns for equities than firms with low P/E ratios, and this effect is not just limited to stocks with small capitalization. According to Ref. [9], small stocks perform better than big stocks. Similarly [10], found that companies that possess a high book-to-market ratio (BMR) tend to outperform those companies that have a low BMR.

However [11], FF3 includes size, BMR (book-to-market ratio), and market risk premium thus to propose FF3FM. Further, it finds that FF3FM describes stock return variability better than CAPM. Similarly [12], find that after incorporating human capital, the performance of CAPM substantially improves from 3% to 75%. [13], introduces the concept of human capital as the source of wealth. Later, the author used the increased in salaries received by labor as a tool to measure human capital. Further, it finds that the single factor model (CAPM) does not account for majority of the variation in excess returns. Contrary to CAPM specification, labor-income betas (β) significantly enhance CAPM performance. Similarly [14], finds association among human capital and stock returns.

Similarly [15], proposed the four-factor model (C4FM), which includes an additional factor that is momentum to the FF3, and found it superior than FF3FM for explaining stock returns variation. [16], argue that the role of human capital in asset pricing varies across business cycle frequencies. They find that human capital is positively related to stock returns at low frequencies, which corresponds to the business cycle, but negatively related at high frequencies, which correspond to monthly frequencies. This suggests that the relationship between human capital and stock returns is complex and may depend on the time horizon and frequency of analysis. Later [17], include human capital factor in CAPM. Moreover, the study concluded that the beta (β) associated with human capital could explain the cross-sectional variations. [18], tested the efficiency of the conditional CAPM with human capital, and found that including rich measurements of human capital (such as the cost and benefit of educational investment and the skill premium) improved the sustainability and performance of the model. [19], proposed a novel method to evaluate the significance of human capital in asset returns. It is concluded that the model effectively accounts for the variation in asset returns. The findings show that it is a significant predictor of asset returns [20].

Later [21], proposed the five-factor model (FF5) and added two additions, namely profitability and investment factor, to FF3. Later [22], expanded the FF5 to six factors by including the momentum factor. Similarly, to this [23], proposed a six-factor asset pricing model (RS6) and added human capital to the FF5. Similarly [24], find that labor-income growth rate statistically captures the variation in asset returns.

The stock market of Pakistan (an emerging country) is particularly important for several reasons. The geopolitical position suggests that if it can achieve political stability and appropriately use its resources, it has a lot of economic potential. As a result, prospective investors are interested in learning about the return on their investment and associated risk [25]. Pakistan has a unique market structure having many but small size and moderately to highly invested firms with lower profitability, low market cap with more trading, and high returns with significant volatility [26]. Moreover, the high degree of concentration in certain sectors such as banking and oil and gas, providing interesting insights into asset pricing studies. Furthermore, Pakistan is still an under-research market, having the potential for researchers to make novel contributions to the field of asset pricing in comparison of other developed markets.

Most importantly, has a long history of political and economic instability that effects the prices of financial assets, making asset pricing more important. Overall, Pakistan's large and growing economy, emerging market status, political and economic reforms, availability of data, and diversity of financial instruments make it an attractive country for asset pricing study.

The importance of asset pricing in Pakistan provided in early literature includes [[27], [28], [29], [30], [31], [32]]. Looking into affirming literature conducted on asset pricing models and the importance of human capital further moves forward the importance of multifactor models. Therefore, there is a need for a study to expand the FF3 by adding human capital as a fourth factor and to test the new proposed model. Therefore, the current study aims to extend the FF3 with an additional factor, that is, human capital, thus to proposed an augmented human capital four-factor model in Pakistan, which represents one of the Asian emerging economies. Similarly, investment on human capital play vital role in predictability of assets returns therefore, it has greater effect on company value [33,34].

This study is constructed on the theory of [3], the Capital market theory of [4], and the market efficiency theory of [35]. According to Ref. [3], who proposed Markowitz's portfolio theory that discusses the construction of optimal portfolios of risky assets. The theory assumes that investors are risk-averse and want to maximize expected returns while minimizing risk. Additionally, the research highlights that investors can achieve their investment goals by creating a diversified portfolio that provides the maximum expected return for a specific risk level or the minimum risk level for a given expected return. Introduced the capital asset pricing model (CAPM), which relies on the principles of Markowitz portfolio theory. According to the CAPM, investors are rewarded for taking on two types of risk: systematic risk, which cannot be eliminated through diversification and is represented by an asset's beta, and unsystematic risk, which can be eliminated through diversification but is not rewarded with compensation. According to the model, expected return of assets is proportionate to its beta, which represents its sensitivity to systematic risk. According to Ref. [35], market efficiency theory holds that financial markets incorporate all publicly available information into the price of securities, this information makes it impossible for investors to consistently achieve returns above the average market returns. Market efficiency is very important for asset pricing models. To hold market efficiency, the CAPM must hold.

Overall, the theory provides a foundation to understand the pricing of financial assets and allows investors to make informed decisions about the allocation of their portfolios.

There are two contributions to our study: First, our study proposes and tests the validity of the augmented four-factor model. We believe that this study is the first to expand on FF3 by adding human capital as a new factor. Second, this study covers an emerging market in Asia that is not much explored in empirical studies on human capital. The findings of this study indicate that human capital significantly explains the predictability in asset returns. Moreover, the augmented HC4FM (human-capital four-factor) is found valid for the prediction of the variation in excess portfolio returns.

The remaining paper is organized as follows. Detailed literature on asset pricing is presented in Section 2. Section 3 covers the research methodology, which consists of portfolio formation, factor construction, and model estimation. Section 4 consists of a statistical analysis of the data followed by time series regression, and Section 5 concludes the article.

## 2 Review of existing literature

In recent times, CAPM has attract the attention of many researcher across the world for investment decision. Similarly, the strengthens of CAPM for explaining the relationship between risk and return has face many criticism [[7], [8], [9],^36^]. Similarly, the arbitrage pricing theory of [7], known for the unidentified factor which ultimately affects stock returns. Later, some known factor were introduced, GDP growth, country inflation and firm's dividend yield. Further, these identified factor were questioned many studies [37]. Generally, references to consumption, 75% of consumption is based on human capital. Thus human capital plays pivotal role in stocks return predictability. Primarily, human capital is important for aggregate wealth [38].

According to Ref. [39], the CAPM premise is open to challenge because it only considers market risk when calculating stock returns. It is further suggested that stock returns rely on factors other than just market premium. Furthermore, it comes to the conclusion that single beta (β) is insufficient to fully explain market returns. Therefore, market, size, and value factor are taken into consideration in FF3FM, which is based on the conventional CAPM. According to Ref. [40], holding periods of three to twelve months’ result in returns. These strategies buy companies that have been performing well and sell stocks that have been underperforming in order to produce substantial gains. Later [15], presented a four-factor model (henceforth C4FM) to replace the FF3FM by adding momentum as a fourth factor, and found that it more superior for explaining the variability in stock returns.

Human capital needs to be viewed as an investment rather than a cost to the company in the knowledge-based economy of today. According to Ref. [18], provides proof that the cross-section of asset returns may be impacted by investments in human capital across various industries. Similarly [41], conducted research on risk variables based on market and accounting factors. It finds that there is a statistically significant correlation between equity return and human capital. [42], carried out research on the FF3. It concludes that the equity return is influenced by size and value premium. Additionally, it provides evidence for the FF3 limited description in Asian markets as a means of elucidating average portfolio results.

According to Ref. [43], beta (β) is not a reliable indicator of stock return. Further, it finds that significant explanatory power exists for the other two factors FF3 in terms of capturing the variation in stock returns. Consequently [44], in a study compared the CAPM efficiency to that of the FF3. The study came to the conclusion that FF3FM explained risk and return variation better than the single-factor model (CAPM). According to Ref. [45], companies offer greater salaries to employees who enhance their human resources, which boosts output and the firm's worth. According to Ref. [46], it is challenging to duplicate and mimic the distinctive qualities of human asset firms. A company with growing human resources can outperform other companies on a long-term basis. Similarly [47], documents that the market cannot predict the accuracy of human assets for small-size firms. Further, it is important to determine the relationship between human assets and firm valuation. [48], find that incorporating human capital in the asset pricing model predicts the variation in stock return along with size and value premium.

In the context of emerging economies such as Pakistan, investors and portfolio managers are urged to search for modified factor models rather than relying on the single-factor or CAPM [49]. Similarly [50], find that the human capital component has a close relationship with asset pricing models. Further, the FF3 was put to the test in the Bangladesh stock market by Ref. [51], it reveals that small businesses with modest market capitalization perform better than large businesses with substantial market capitalization. Similarly, to this, a corporation reporting low profitability will have a high BMR. Additionally, the FF3 provided less insight into stock returns in the Bangladesh stock market. Numerous researchers fiercely contest and condemn the FF3for its inability to provide an adequate explanation [[52], [53], [54], [55]].

Later [21], proposed FF5 that included market, size, and value premium in addition to profitability and investment factor. The FF3 is determined to be less inferior to the FF5. The FF5 was later found to be unable to account for the variation in stock return in numerous research conducted around the world [56,57]. Further, it justifies the need for other factors to include and proposed new robust model. HC (human capital) plays vital role for capturing the variation in asset returns. Similarly, companies that have low labor growth perform better than those with high labor growth [58]. Similarly [59], used the GRS (Gibbons Ross and Shanken's) test to evaluate the efficacy of the FF3 and FF5. The author contends that the findings of the GRS test for Fama-French models are insufficient. This research also comes to the conclusion that there is still a global effort to create the best asset pricing models. Consequently [60], also discovers that the FF5 is ineffective for identifying long-term abnormalities. Similarly [61], finds that among other, market factor is very important for capturing the predictability in assets returns.

In similar vein [1], show that the FF5 includes human capital in the instance of India and that this component plays an important role for forecasting equity returns. Similarly [23], introduce the FF5 with the inclusion of human capital factor and put forward a novel six-factor asset pricing model (RS6). The study concludes that the human capital factor is a significant explanatory variable for the observed portfolio returns variations. In the Indonesian stock market from 2015 to 2019 [62], tested the RS6 and concludes that the human capital, market, size, value, profitability, investment factors have a significant effect on returns. [63], in the study look at how human capital affects corporate valuation. It concludes that disregarding human resources when valuing a corporation could result in a significant issue. [64], investigated the flaw in FF3 methodology. The authors present evidence of a strong link between the variables.

Similarly [65], document that the quality improvement of skilled labor through education will help to expand outward investment in the country. Further, it finds that investment in human capital have significant impact on country economic development. Later [24], examined the effect of the labor-income-growth rate on stock returns. The authors find that labor-income-growth rate statistically predict the variability in asset returns. The available research demonstrates that human capital has an impact on stock returns all around the world.

Consequently [66], examined how FF3 and FF5 fared during COVID-19 and other crises that occurred in both emerging and established economies over the previous two decades. The author concludes that the FF5's performance is comparable to that of the industrialized market. However, the authors contend that improving asset pricing models by including more variables in regression will result in more trustworthy results. Therefore, a thorough investigation is required to determine how the FF3 and human capital affect stock returns in Pakistan's equities market. The influence of human capital has not previously been studied, nor is it taken into account in asset pricing models, particularly the FF3 used in Pakistan. As a result, by creating a four-factor model based on enhanced human capital, this study closes a gap in the literature.

### 2.1 The hypothesis of the study

H1 Human capital (Labor-income-growth) significantly explains variations in portfolio returns

H2 Four-factor model is a valid model for explaining time series variations in portfolio returns.

## 3 Methodology

### 3.1 Data and variables

We collected monthly share price data for a sample of 164 non-financial companies listed on the Pakistan Stock Exchange (PSX) during the period of July 2010 to June 2020. The monthly share price data was obtained from a business recorder website, while the data for three factors (namely size, value factor, and human capital factor) were sourced from the Balance-sheet-analysis (BSA) report published by the State Bank of Pakistan (SBP) for non-financial companies during the period of 2010–2019. Further, we calculate market capitalization proxy for size, the BMR (book-to-market ratio) for the value factor, and the labor income growth rate as a proxy for the human capital factor. The three-month T-bill rate is used in this study as a stand-in for the risk-free rate (R_f_), and the KSE-100 index adjusted closing return is used for the calculation of market return (R_m_). This study used the approach of [1,[67], [68], [69]], to calculate human capital, which is calculated as (LI_t_-LI_t-1_/LI_t-1_), where LI_t_ denotes labor income for the reference year with time t.

### 3.2 Fama and Macbeth's (1973) regression

In order to examine the validity of the proposed 4-factor model, this study utilizes the approach of [70], two-pass time-series regression (FMB), which is a popular and widely-used method for testing asset pricing models, having been cited in over 18,365 academic research studies. FMB calculates the risk premium and Betas (β) for all risk factors that could potentially affect the asset values.

FMB have three main implications. The first is that the association between risk and return is linear. The second is that the beta is a measure of risk for a particular asset in the portfolio. The third is a condition where there is risk aversion, higher risk should result in higher returns. Later on, a two-step estimation approach for portfolio return was put forth by FMB. During the initial phase, the betas (β) for each portfolio are computed for time t. In the subsequent stage, the time series from the first phase is evaluated by performing a regression analysis between the portfolio's return and the betas (β) estimated previously.

Equation (1) represents FMB first-pass regression

(1) $${Ri}_{t}=\beta_{0}+\beta_{1}\left({MKT}_{t}\right)+\beta_{2}\left({SMB}_{t}\right)+\beta_{3}\left({HML}_{t}\right)+\beta_{4}\left({LMH}_{t}\right)+e_{t}$$

Equation (2) represents FMB second-pass regression

(2) $${Ri}_{t}=\beta_{0}+\beta_{1}\left({\beta MKT}_{t}\right)+\beta_{2}\left({\beta SMB}_{t}\right)+\beta_{3}\left({\beta HML}_{t}\right)+\beta_{4}\left({\beta LMH}_{t}\right)+e_{t}$$

In Eq (1) and Eq (2) $\beta_{0}$ represent intercept term, whereas ${{Ri}_{t}=(R}_{it}-R_{ft}$ represent the portfolio returns over the risk-free rate, ${MKT}_{t}$ represent the market premium, ${SMB}_{t}$ represent size premium, ${HML}_{t}$, value premium, ${LMH}_{t}$**,** labor-income-growth rate and $e_{i}$ represent error term. Whereas the subscript i and t represent the firms and time period.

### 3.3 Construction of the factors

This study constructs a set of eight portfolios using sorting techniques. Selected samples are divided into small and large companies based on market capitalization for size portfolios. For value portfolios, the size-sorted portfolios were further distributed into two subgroups based on the book-to-market ratio (BMR) those with low BMR and those with high BMR. Subsequently, the value-sorted portfolios were further divided into two sub-groups, based on the labor income growth rates of the companies, into those with low growth rates and those with high growth rates. We use the method proposed by Ref. [11], for factor calculation such as SMB, HML, and LMH.

**Size Premium (SMB)** = 1/4* [(SLLo-BLLo) + (SLHi-BLHi) + (SHLo-BHLo) + (SHHi-BHHi)]

**Value Premium (HML)** = 1/4* [(SHLo-SLLo) + (SHHi-SLHi) + (BHLo-BLLo) + (BHHi-BLHi)]

**Human capital (Labor-income-growth) (LMH)** = 1/4* [(SLLо-SLHі) + (SHLо-SHHі) + (BLLо-BLHі) + (BHLо-BHHі)].

In the above equations, the SMB factor signifies the returns of small companies minus the returns of large firms and emulates the company risk related to size. Similarly, the HML factor represents the returns of high book-to-market ratio (BMR) firms minus the returns of low BMR firms and mirrors the risk linked to company value. Likewise, the LMH means returns of low labor income growth rate firms minus returns of high-labour-income-growth rate firms and mimics the risk associated with company human capital. .

**Table 1:** Summary Statistics of portfolios.

	Mean	SD	Min	Max
SLLo	0.026	0.103	−0.189	0.397
SLHi	0.019	0.068	−0.125	0.199
SHLo	0.023	0.101	−0.158	0.353
SHHi	0.02	0.078	−0.15	0.261
BLLo	0.022	0.084	−0.146	0.391
BLHi	0.017	0.071	−0.123	0.281
BHLo	0.026	0.083	−0.151	0.307
BHHi	0.017	0.073	−0.244	0.227

Where SD= Standard Deviation, Max, Min = Maximum and Minimum.

**Table 2:** Summary Statistics of risk factors.

	Mean	SD	Min	Max
MKT	−0.018	0.059	−0.275	0.129
SMB	0.001	0.03	−0.083	0.084
HML	−0.002	0.028	−0.084	0.089
LMH	0.0061	0.039	−0.088	0.119

Refers to Table 1.

**Table 3:** Pearson Correlation of risk factors.

	MKT	SMB	HML	LMH	VIF
MKT	1				1.016
SMB	−.063	1			1.154
HML	−.244**	−.048	1		1.093
LMH	.212*	.204*	−.358**	1	1.193

**' * indicate correlation at 1%, 5% level of significance respectively.

**Table 4:** First-pass regression (augmented human capital four-factor model).

	Intercept	MKT	SMB	HML	LMH	Adj. R^2^	F-stat	*P*-value
SLLo	−0.01 (−1.426)	0.248 (2.135)**	1.002 (4.508)**	−0.393 (−1.583)	1.359 (7.389)***	0.516	32.772***	0.000
SLHi	−0.01 (−1.651)*	0.132 (1.26)	0.785 (3.921)**	0.043 (0.193)	0.1001 (0.604)	0.111	4.741**	0.001
SHLo	−0.012 (−1.92)*	0.172 (1.638)*	0.936 (4.666)**	1.875 (8.342)*	1.673 (10.055)*	0.592	44.265***	0.000
SHHi	−0.01 (−1.494)	0.132 (1.183)	0.854 (4.001)**	0.964 (4.036)**	0.385 (2.179)**	0.213	9.073**	0.000
BLLo	−0.01 (−1.889)*	0.132 (1.2004)	0.854 (−0.183)	0.964 (0.669)	0.385 (6.803)***	0.312	14.492**	0.000
BLHi	−0.011 (−1.633)*	0.208 (1.855)*	−0.017 (−0.082)	0.51 (2.118)**	0.413 (2.322)**	0.058	2.838**	0.027
BHLo	−0.007 (−1.104)	0.128 (1.123)	−0.32 (−1.465)	0.846 (3.457)**	1.267 (6.989)*	0.298	13.635**	0.000
BHHi	−0.011 (−1.701)*	0.211 (1.957)**	−0.0453 (−0.219)	0.971 (4.207)**	0.618 (3.618)**	0.161	6.713**	0.002

**Table 5:** Two-pass Fama-Macbeth's (1973) regression.

	Intercept	MKT	SMB	HML	LMH	Adj. R^2^	F-stat	*P*-value
SLLo	−0.189 -(1.075)	0.126 (1.097)	0.034 (0.509)	0.023 (0.718)	0.106 (1.127)	−0.017	0.712	0.615
SLHi	−0.073 (-1.438)	−0.097 (-1.49)	0.139 (1.989)	0.125 (2.965)	0.081 (0.995)	0.073	2.308	0.052
SHLo	−0.454 (-1.763)	−0.078 (-0.878)	0.158 (1.443)	0.112 (2.241)	0.104 (0.99)	0.069	2.248	0.057
SHHi	0.488 (0.296)	−0.012 (-0.136)	−0.064 (-1.363)	0.035 (0.944)	−0.073 (-0.973)	−0.031	0.498	0.776
BLLo	−0.087 (-0.854)	0.021 (0.267)	0.085 (0.989)	0.065 (1.781)	0.081 (0.998)	0.017	1.289	0.277
BLHi	−0.031 (-0.921)	0.014 (0.197)	0.041 (0.602)	0.012 (0.326)	0.066 (0.87)	−0.014	0.757	0.583
BHLo	0.363 (0.202)	−0.009 (-0.122)	−0.019 (-0.679)	0.018 (0.316)	0.013 (0.225)	−0.012	0.787	0.562
BHHi	−0.043 (1.069)	0.034 (0.374)	0.028 (0.401)	0.026 (1.027)	0.024 (0.376)	−0.016	0.733	0.6

Note: Refers to Table 4.

## 4 Empirical results

This table summarizes the eight portfolios sorted on size, BMR ratio, and labor-income-growth rate. The size-sorted portfolios show that the four small-sized portfolios have higher risk and return than comparable large-size portfolios (SLLo, SLHi, SHLo, SHHi are greater than BLLo, BLHi, BHLo and BHHi). Therefore, small size portfolio earns higher returns than big stocks portfolios because small portfolios are riskier than larger stock portfolios.

Further, for value sorted portfolios, it is indicated that high BMR portfolios considerably report higher risk and return than the portfolio with low BMR, i.e. (risk and returns of SHLo, SHHi, BHLo BHHi report higher risk and returns of SLLo, SLHi, BLLo, and BLHi). Further, it is evident that portfolios with high BMR are riskier and earn higher returns than the portfolio with low BMR. Furthermore, the study finds that the portfolio with a low labor-income growth rate exhibits a notably higher return and risk compared to the portfolio with a high labor-income growth rate when sorted based on labor-income growth rate i.e. (SLLo, SHLo, BLLo, BHLo are greater than SLHi, SHHi, BLHi and BHHi). Additionally, it is clear that portfolios with low rates of labor income growth are riskier and earn higher returns than portfolios with high rates of labor income growth.

This table shows the summary statistics of MKT, SMB, HML, and LMH**.** It shows that MKT has a mean value of −0.018, the SMB is 0.001, the HML is −0.002, and the LMH is 0.0061. The negative value of MKT for the Pakistan equity market is found to be similar to the results of [71]. The authors document that very high standard deviation and negative market value show higher volatility in returns.

This table shows the correlation matrix of the risk factor. The market premium and SMB is found to be negatively correlated according to previous literature, but the relation is found statistically insignificant, while HML is also found negatively correlated with market premium and size factor, but the relation is found statistically significant at 1% significance level. Whereas, at a 5% significance level, the LMH factor exhibits a positive correlation with the market premium and a negative correlation with both the size and value factors. Moreover, the values are found in the tolerable limit, and VIF values are also found in the limit. Indicated that no problem of Multicollinearity exists among explanatory variables. Multicollinearity is a concern if VIF exceeds five and the tolerance value falls below 0.20 [72].

This Table summarize the regression results of the four-factor model, which includes the market (MKT), size (SMB), value (HML), and labor-income-growth rate (LHM) factors. It shows that the market premium has a positive and statistically significant effect. Whereas, for SMB, it is discovered that all small portfolios exhibit positive and statistically significant results, while big portfolios exhibit insignificantly positive and negative results. This finding suggests that investors should take into account the firm size based on market capitalization when investing in firms. Previous studies have indicated that small companies tend to generate higher returns compared to larger firms, thereby compensating investors for the additional risk involved in investing in smaller portfolios. Moreover, for both small and large companies with low BMR, the expected outcome of the HML factor is reported to be a negative coefficient that lacks statistical significance. However, it is advantageous and significant for both small and big portfolios with high BMR, suggesting that investors should invest in the stock's high BMR since the company's high MBR value outperforms the stock's low book-to-market value. For small and large portfolios sorted on labor income-growth rate, the estimated result of the LMH or human capital factor is positive and statistically significant (aside from SLH), indicating that human capital statistically explain the predictability in portfolio-returns. The augmented HC4FM (human-capital-four-factor) adjusted R-square has a range of 5.8%–59.2%, indicating that the probability of variation in portfolio returns is explained by these four factor. The probability values and F-statistics for each portfolio were found to be statistically significant, the analysis demonstrates that these four factors have a substantial impact on portfolio returns.

This table summarize the two-pass regression estimation output of the augmented human capital four-factor model. The result is obtained by performing a 36-months rolling window regression technique. In the first step, we regress the estimated four factors (MKT, SMB, HML, LMH) for each of the portfolios. Then we regress the estimated beta's (β) obtained from first-pass regression to predict the future returns for each portfolio based on historical beta's (β) values. The estimated result from the table is found to be statistically insignificant (*P*-value) for all of the portfolios, namely MKT, SMB, HML, and LMH. The adjusted R-squared of small to large portfolios is observed to be low. Additionally, the probability value of the F-statistic and its p-value are found to be statistically insignificant for all portfolios. These findings suggest that past betas (β) cannot be utilized to forecast future returns. These results are consistent with the efficient market theory, which emphasizes that in an efficient market, equities will be fairly valued and reflect all available information accurately. The stock price as it stands right now includes all information that could be used to forecast stock performance.

The Efficient Market Hypothesis (EMH) was developed based on the fair game model and random walk theory, as proposed by Refs. [35,73]. Subsequently, the EMH classified efficient capital markets into three categories, namely weak, semi-strong, and strong form. The weak form suggests that prices accurately reflect the information contained in the price history. The semi-strong form states that market prices reflect all publicly available information that is relevant, while the strong form argues that prices incorporate any information known to any participant. As a result, the EMH demands that an efficient market be characterized as one in which relying on available knowledge to trade does not produce an extraordinary profit. Therefore, a market can only be considered efficient if a return model is proposed. As a result, market testing has changed to include evaluations of both asset pricing models and market behaviour. One important implication of the EMH is that stock prices change arbitrarily and in an unpredictable way or that they follow a random walk. Prices must vary in reaction to new information if they are to move at all if they are established at their current levels based on all the information currently available. This means that price changes must essentially be unexpected.

## 5 Discussion

Our findings align with those of previous studies such as [11], who contend that the market risk factor is necessary to account for returns beyond the risk-free rate, but it is inadequate to account for the variation in excess stock returns. Furthermore, they observed that the slope of the SMB is more significant for small stocks than for large stocks. Stocks with a higher BMR than those with a lower one are also more sensitive to HML.

Similarly [74], finds that small stocks outperform large stocks in terms of return, while stocks with a high BMR of equity outperform those with a low BMR of equity. Similar findings were report by Ref. [75], that in Pakistani equity market, small-size portfolio returns are higher than returns of large-size portfolios. Although [1], conclude that in the context of India, earlier studies mainly ignore the human capital to include in models (asset-pricing). Similarly [76], evaluated the FF3 before and after the 2008 financial crisis. The authors find that the FF3 is effective at explaining variation in asset yields both before and after the financial crisis.

According to Ref. [48], asset pricing model with human capital as a factor helps to predict stock return variance together with size and value premium. Similar to Ref. [41], studied that market and accounting variables depend on risk. The evidence suggests a significant statistical relationship between human capital and equity returns. Similarly, human capital paly vital role in assets pricing. Further, human capital statistically captures the predictability in assets returns [33,34,77].

According to Ref. [65], increasing the quality of skilled workers will increase foreign investment in the nation. According to Ref. [24], in the context of emerging economies, i.e., Pakistan, portfolio managers and investors may benefit from considering the potential impact of the human capital factor in the asset pricing model. Which is still a relatively new concept, and it's great to see more research exploring its impact on stock returns. By incorporating human capital, investors may be better able to account for the value of a company's workforce and their ability to generate profits in the future. This could lead to more accurate pricing of stocks and potentially better investment decisions. In the FF3, an enhanced four-factor model of human capital is found effective for explaining variation in assets returns along with other factor SMB (size) and HML (value).

## 6 Conclusion

The objective of the study is to test the validity of a four-factor model in Pakistan-an extension of the Fama-French three-factor model that includes human capital as a factor. The study forms eight portfolios based on size, value, and human capital factors and collects data of 164 non-financial firms listed on the PSX from July 2010 to June 2020. The two-pass time series regression method proposed by FMB is used to test the legitimacy of the proposed four-factor model. This study finds that size and value premiums have an impact on excess portfolio returns. According to the literature, small stocks compensate investors for taking on more risk by paying out higher returns than big stocks. Similarly, high BMR stocks report higher returns than low BMR stocks. Similarly, company investment on human capital replicate positive responses to company value which in terms helps investors to make rational decisions [1].

The neoclassical growth theory contends that human capital is a production input factor, but it treats it similarly to physical capital in that it has diminishing returns. Economic growth is not possible without technological innovation. According to the endogenous growth hypothesis, education and human capital are distinct, significant factors in production, yet the knowledge-based economy has a multiplier and spillover effect that promotes economic growth. Dividing labor based on skill and allowing agents to choose their level of expertise could help us better assess the value of human capital investments. Last but not least, the importance of a competitive salary across industries may have an impact on the cross-section of asset returns. The demand for labor in that business will rise as a result of a favorable technology shock, raising wages throughout the economy and impacting the returns in all other industries. Thus, labor markets significantly influence how risky firms' cash flows are, and a better understanding of this influence can help us better specify the cross-section of asset returns.

The study's findings unequivocally support the significance of human capital in corporate value and underestimating it could have catastrophic consequences. Investors who want to profit from investment premiums should take into account the firms' use of human resources when making investment decisions. Investors will also have more informational advantages over rivals if they take human asset investment into account when valuing firms. According to the study, human capital is a key factor in estimating returns, which has a significant implication on public policy content.

It is suggested that future research should take into account the human capital factor when constructing asset-pricing models. In addition, this study encourages investors to deliberate HC (human-capital) in their rational decision-making. When selecting investment options, investors should consider human capital as well as other factors such as size (SMB) and value (HML) premiums. The empirical findings of this study encourage academics and all sorts of investors to take human capital into account when developing models to determine the required return on portfolios returns.

Further, we observed a few limitations of this study. First, we did not construct traditionally 5 × 5 sorted portfolios. Second, this study only constructs a set of eight portfolios. Further, research can be carried out by taking larger data sets to construct portfolios by according to Refs. [11,21], research methods. Such a study can also be carried out by examining the efficiency of asset-pricing models in the COVID-19 outbreak in the emerging market of Pakistan. Similarly, future studies may also conduct a comparative study by comparing the proposed 4-factor model with other alternative asset pricing models.

## Author contribution statement

Naveed Khan: Conceived and designed the analysis, Analyzed and interpreted the data, and Wrote the paper.

Hassan Zada: Contributed analysis tools or data, Analyzed and interpreted the data.

Shakeel Ahmed: Analyzed and interpreted the data and Wrote the paper.

Fayaz Ali Shah: Contributed reagents, materials, analysis tools or data.

Shahid Jan: Contributed analysis tools or data, and proof read the paper.

## Data availability statement

This study uses monthly share price data of 164 non-financial companies listed on the Pakistan Stock Exchange, officially known as the KSE-100 Index, from business records webiste for the period July 2010 to June 2020. Data used for factor calculations are extracted from the 2010–2019. Balance Sheet Analysis (BSA) report published by the State Bank of Pakistan (SBP). This study obtains data from selected non-financial companies and calculates market capitalization (a proxy for size).

## Declaration of competing interest

The authors declare that they have no known competing financial interests or personal relationships that could have appeared to influence the work reported in this paper

## Appendix A

Portfolios and its Abbreviation.

## Abbreviations

SLLo: Small cap, low BMR and low-income-growth rate firms
SLHi: Small cap, low BMR and high-income-growth rate firms
SHLo: Small cap, high BMR ratio and low-income-growth rate firms
SHHi: Small cap, high BMR and high-income-growth rate firms
BLLo: High cap, low BMR and low-income-growth rate firms
BLHi: High cap, low BMR and high-income-growth rate firms
BHLo: High cap, high BMR and low-income-growth rate firms
BHHi: High cap, high BMR and high-income-growth rate firms
